# INFLAMMATION’s cognitive impact revealed by a novel “Line of Identity” approach

**DOI:** 10.1371/journal.pone.0295386

**Published:** 2024-03-22

**Authors:** Donald R. Royall, Raymond F. Palmer

**Affiliations:** 1 Department of Psychiatry and Behavioral Science, The University of Texas Health Science Center, San Antonio, Texas, United States of America; 2 Department of Medicine, The University of Texas Health Science Center, San Antonio, Texas, United States of America; 3 Department of Family and Community Medicine, The University of Texas Health Science Center, San Antonio, Texas, United States of America; 4 The Glenn Biggs Institute for Alzheimer’s and Neurodegenerative Disease, The University of Texas Health Science Center, San Antonio, Texas, United States of America; Fondazione Don Carlo Gnocchi, ITALY

## Abstract

**Importance:**

Dementia is an “overdetermined” syndrome. Few individuals are demented by any single biomarker, while several may independently explain small fractions of dementia severity. It may be advantageous to identify individuals afflicted by a specific biomarker to guide individualized treatment.

**Objective:**

We aim to validate a psychometric classifier to identify persons adversely impacted by inflammation and replicate it in a second cohort.

**Design:**

Secondary analyses of data collected by the Texas Alzheimer’s Research and Care Consortium (TARCC) (N = 3497) and the Alzheimer’s Disease Neuroimaging Initiative (ADNI) (N = 1737).

**Setting:**

Two large, well-characterized multi-center convenience samples.

**Participants:**

Volunteers with normal cognition (NC), Mild Cognitive Impairment (MCI) or clinical “Alzheimer’s Disease (AD)”.

**Exposure:**

Participants were assigned to “Afflicted” or “Resilient” classes on the basis of a psychometric classifier derived by confirmatory factor analysis.

**Main outcome(s) and measure(s):**

The groups were contrasted on multiple assessments and biomarkers. The groups were also contrasted regarding 4-year prospective conversions to “AD” from non-demented baseline diagnoses (controls and MCI). The Afflicted groups were predicted to have adverse levels of inflammation-related blood-based biomarkers, greater dementia severity and greater risk of prospective conversion.

**Results:**

In ADNI /plasma, 47.1% of subjects were assigned to the Afflicted class. 44.6% of TARCC’s subjects were afflicted, 49.5% of non-Hispanic Whites (NHW) and 37.2% of Mexican Americans (MA). There was greater dementia severity in the Afflicted class [by ANOVA: ADNI /F(1) = 686.99, p <0.001; TARCC /F(1) = 1544.01, p <0.001]. “INFLAMMATION” factor composite scores were significantly higher (adverse) in Afflicted subjects [by ANOVA in ADNI /plasma F(1) = 1642.64, p <0.001 and in TARCC /serum F(1) = 3059.96, p <0.001]. Afflicted cases were more likely to convert to AD in the next four years [by Cox’s F, ADNI /plasma: F (252, 268) = 3.74 p < 0.001; TARCC /serum: F (160, 134) = 3.03, p < 0.001 (in TARCC’s entire sample), F (110, 90) = 4.92, p <0.001 in NHW, and F(50, 44) = 2.13, p = 0.006 in MA]. The proportions converting were similar among afflicted NHW in both cohorts /biofluids but MA exhibited a lower risk (7% in TARCC /serum at 48 months).

**Conclusions and relevance:**

Our inflammation-specific psychometric classifier selects individuals with pre-specified biomarker profiles and predicts conversion to “AD” across cohorts, biofluids, and ethnicities. This algorithm might be applied to any dementia-related biomarker making the psychometric estimation of individual biomarker effects feasible without biomarker assessment. Our approach also distinguishes individuals resilient to individual biomarker effects allowing for more accurate prediction and precision intervention.

## Introduction

Dementia care demands a growing fraction of healthcare resources in the United States (US). In 2021, 6.2 million (11.3%) of Americans aged 65 years and older were estimated to have a dementia which may cost an estimated $355 billion [1]. By 2050, these annual costs could rise to $1.1 trillion. From 2000–2019, US deaths from dementia rose 145% while deaths from heart disease decreased. Regardless, after adjusting for population growth, the number of fellowship-trained geriatric psychiatrists fell 18% between 2001–2018 [2]. Clearly, the future of dementia care will fall to non-fellowship trained geropsychiatric providers, a scenario envisioned by *The Geriatric Imperative*, as early as 1981 [3].

Those providers will likely consult algorithms derived from informatic classifiers. The National Alzheimer’s Program Act (NAPA) has called for research on such diagnostic strategies and many groups have responded by proposing novel biomarkers [4]. Regardless, there are limits to the widespread deployment of dementia-related imaging and cerebrospinal fluid (CSF) biomarkers. Blood-based proteins have been associated with dementia and offer more easily acquired classifiers. However, their replication has proven to be difficult.

This state of affairs may indicate a misdirection in our conceptual approach to dementia. The dementia status of individuals is likely to be “overdetermined” i.e., to arise from the aggregated effects of multiple independent processes [5]. Consequently, no single biomarker is likely to explain a large fraction of dementia severity and meaningful improvements may require interventions on multiple biomarker-related processes and /or focused deployment in highly afflicted fractions (i.e., “Precision Medicine”) [6].

We have been using theory-driven confirmatory bifactor analysis (CFA) in a Structural Equation Model (SEM) framework to refine dementia’s assessment [7]. By that approach, we have developed a dementia-specific phenotype, i.e., “δ”, representing the shared variance between a cognitive battery and a measure of Instrumental Activities of Daily Living (IADL). δ’s rationale is that dementia’s only essential feature need be a disabling cognitive impairment. δ distinguishes functionally salient cognitive impairment (FSCI) from cognitive impairment *per se*.

As dementia’s essential phenotype, δ is agnostic to etiology and cannot discriminate between any two dementing conditions. Gavett et al. [8] showed δ to have an area under the Receiver-Operating Characteristic curve (AUC /ROC) = 0.96 for All-Cause dementia in data from the National Alzheimer’s Coordinating Center (NACC) (± 25K subjects). John et al. [9], subsequently showed it to be incapable of distinguishing any two dementias by etiology (i.e., they are all dementias and δ is their essential feature). In contrast, the cognitive changes that do distinguish individual dementing conditions are revealed *ipso facto* to be inessential to dementia, and irrelevant to functional disablement. It is imprecise therefore to think of cognitive impairment *per se* as evidence of dementia severity.

δ can be estimated from a wide range of indicators necessitating the distinction among each instance as a “δ homolog”. In genetics, a homolog is a gene descended from an ancestral gene in the same species and which retains the original’s function. 17 δ homologs have been validated to date [10,11]. Each is derived from a unique set of cognitive and /or functional indicators.

As for any latent variable, δ can be “reified” as a composite factor score. When a δ homolog is reified, it becomes a “d-score” which can be applied to individuals as a continuously distributed measure of dementia severity. As a continuous measure, the d-score indicates the severity of FSCI at any point in dementia’s evolution, even among cognitively normal controls (NC). Only a reified d-score can be submitted to ROC. All d-scores published to date are strongly associated with dementia severity as determined by clinicians [i.e., via the Clinical Dementia Rating Scale (CDR) [12] “Sum of Boxes” (CDR-SB) [13] and achieve high AUCs for AD’s discrimination from controls.

Either the d-score or its latent homolog can be leveraged to identify biomarkers specific to the dementia syndrome and having functionally salient cognitive effects. We have identified several serum protein biomarkers of δ homologs in the Texas Alzheimer’s Research and Care Consortium (TARCC) [14,15]. We have also published the serum protein mediators of individual dementia risks, including age, depressive symptoms, and the apolipoprotein (APOE) ε4 allele [16–18]. Regardless, TARCC has its limitations. No imaging is available and its protein biomarkers have been obtained in serum. Biomarker associations may be impacted by the biofluid in which they are measured and are notoriously difficult to replicate [19].

However, the Alzheimer’s Disease Neuroimaging Initiative (ADNI) has a cognitive battery that overlaps substantially with TARCC’s, and both studies have deployed similar blood-based biomarker panels processed by a common vendor. Thus, ADNI–TARCC can be used to replicate each other’s biomarker findings. To that end, we have constructed and validated the “d TARCC to ADNI” (dT2A) homolog [20] and have replicated some of TARCC’s blood-based biomarker associations with δ across cohorts and biofluids. [15,21,22] The dT2A homolog fits the data of both datasets well [20]. It has acceptable factor determinancy by Grice’s method [23] and is strongly associated with the CDR-SB [i.e., TARCC: r = 0.99, p <0.001; ADNI: r = 0.96, p <0.001]. dT2A achieves very high AUC in both cohorts [i.e., TARCC: AUC = 0.981 (0.976–0.985); ADNI: AUC = 0.988 (0.983–0.993)].

We recently demonstrated our ability to identify individuals who have been converted to clinical dementia by any single δ-related risk factor. However, such “single-origin” conversions appear to be rare. [24] Instead, dementia’s overdetermined nature will demand that psychometric measures, such as the d-score, distinguish individuals who have been adversely impacted by the specific biological process being targeted for intervention. The recent Food and Drug Administration (FDA) approvals of the anti-amyloid medications and Lecanumab make this a pressing issue. The present analysis is intended demonstrate the potential utility and generalizability of our approach.

We have previously described a latent “INFLAMMATION” construct indicated by nine pro- and anti-inflammatory proteins measured in serum (TARCC) or in plasma (ADNI). Those proteins were originally selected for their associations with interleukin 10 (IL-10). We have associated the INFLAMMATION factor in both biofluids with d-scores in each cohort [21]. Anti-inflammatory agents have failed to improve dementia severity in recent clinical trials. However, those trials involved unselected cases [25]. We propose instead to use a novel “Line of Identity” (LOI) algorithm to select inflammation- “afflicted” cases.

Our LOI algorithm can identify individuals who are adversely impacted by any δ-related biomarker, potentially at any stage of dementia severity. Such persons could then be triaged for individually directed interventions against the targeted biomarker or risk factor. In this approach, a δ homolog is constructed and then adjusted for the biomarker(s) of interest, e.g., the INFLAMMATION construct in this analysis. The adjusted and unadjusted factors are reified as d-scores. Those d-scores can then be correlated, and a scatterplot obtained from their association. A LOI can be drawn through the scatterplot. Because the two composites differ only by the biomarker’s effect, in this case INFLAMMATION’s, any deviation from the LOI must reflect the biomarker’s unique impact on dementia severity as measured by δ. The LOI can then be used to divide the sample into participants presenting above vs. below the LOI.

For this analysis, we again use the dT2A homolog. When a d-score, like dT2A, is inversely scaled relative to cognitive performance, higher scores reflect greater levels of dementia severity [20]. Thus, cases presenting below the LOI improve their d-scores when the effect of INFLAMMATION is covaried out of δ’s estimation. They can be said to have been “adversely impacted” by INFLAMMATION. Cases presenting at or above the LOI are then “Resilient”. To assess generalizability, we will replicate our LOI algorithm in two cohorts /biofluids (i.e., ADNI /plasma and TARCC/ serum).

Finally, we have found cross ethnic differences in the associations between serum levels of the individual cytokine proteins that make up our INFLAMMATION construct and dementia severity as measured by an ethnicity-equivalent δ homolog [14,26]. We will attempt to replicate out LOI findings in two ethnic samples, non-Hispanic Whites (NHW) and Mexican Americans (MA).

We predict that the inflammation-Afflicted groups in both cohort’s /biofluids, ethnicities will have adverse levels of inflammation-related blood-based biomarkers, greater dementia severity and greater risk of prospective conversion to clinical “AD” from non-demented states. We make no representation as to the neuropathological validity of “AD”‘s clinical diagnosis, as it remains to seen if INFLAMMATION acts on δ through AD-specific neurodegenerative changes or independently of them.

## Methods

### Subjects

#### Ethics approval and consent to participate

This is a secondary analysis of deidentified data provided by the Alzheimer’s Disease Neuroimaging Initiative (ADNI) and the Texas Alzheimer’s Research and Care Consortium (TARCC). These are multi-center studies. Each cohort’s protocol was approved by each site’s respective Institutional Review Boards (IRB). Informed consent was obtained from all participants (or their legally authorized proxies) before data collection began.

#### ADNI

ADNI is a well characterized longitudinal convenience sample developed to validate the magnetic resonance imaging (MRI), positron emission tomography (PET), CSF, and genetic biomarkers of AD [27]. We used all 1737 participants of ADNI-1, ADNI-2, and ADNI-GO although only N = 809 had plasma protein biomarkers.

#### TARCC

TARCC is a large (N = 3497), well characterized, ethnically diverse convenience sample with annual longitudinal follow-up [28]. Each participant underwent a standardized annual examination that included a medical evaluation, neuropsychological testing, and clinical interview. Categorical clinical diagnoses of Alzheimer’s disease (AD), “Mild Cognitive Impairment”(MCI), or NC were established through the consensus of clinicians and psychometric raters. N = 1230 (35.7%) were MA. N = 2215 (64.3%) participants were NHW.

### Clinical variables

#### dT2A, a δ homolog for ADNI

We used the dT2A homolog [20]. dT2A has been engineered specifically to facilitate biomarker replications between the TARCC and ADNI. It is indicated by observed cognitive measures that are common to both studies, including the Boston Naming Test (BNT) [29], Category Fluency (Animals) from the Consortium to Establish a Registry for Alzheimer’s Disease (CERAD) battery [30], Logical Memory I (LMI) and II (LMII) from the Wechsler Memory Scale [31], the Mini-Mental Status Examination (MMSE) [32], and Trail-Making Part B (Trails B) [33].

*dT2A’s Target Indicators*. In TARCC, we used the informant-rated Older Adults Resources Scale (OARS) measure of instrumental activities of daily living (IADL) [34] as dT2A‘s target indicator. Unfortunately, IADL is not available in ADNI, and so the Functional Assessment Questionnaire (FAQ) [35] is used instead. The FAQ is commonly used in dementia studies [36] and has been successfully incorporated into δ homologs by other investigators [8,9].

#### Blood-based biomarkers

Blood-Based Biomarkers were processed in both studies by a common vendor (Rules-Based Medicine (RBM) in Austin, TX). RBM conducted multiplexed immunoassay via their human multi-analyte profile (human MAP). The data were standardized to a mean of zero and unit variance. The original reflective INFLAMMATION factor [21] was indicated by Alpha-2 macroglobulin (A2M), interleukins 3 (IL-3), 10 (IL-10), and 13 (IL -13), pancreatic polypeptide (PPP), prolactin (PRL), serum amyloid protein (SAP), Tumor necrosis factor alpha (TNFa), thrombopoietin (THPO), and von Willebrand factor (vWF) as previously described [21].

#### Competing dementia risks

Age, an APOE ε4 (+) genotype, depressive symptoms and gender are independently associated with δ. However, the effect of INFLAMMATION on δ is independent of their effects [21]. These dementia risk factors can therefore be considered as competing determinants of dementia severity as measured by δ.

#### Age

Age was calculated in years from date of birth in both datasets.

#### APOE genotyping

APOE genotyping was conducted in both datasets using standard polymerase chain reaction (PCR) methods. APOEε4 status was coded dichotomously based on the presence or absence of an ε4 allele.

#### Depression

Depressive symptoms were assessed in both studies by the Geriatric Depression Scale (GDS) [37]. GDS scores range from zero-30. Higher scores are worse. The GDS is valid in persons with dementia [38].

#### Gender

Self-reported gender was scored dichotomously with female = 0 and male = 1.

### Statistical analyses

#### Approach

We first tested our LOI algorithm in ADNI and then replicated it in TARCC. The first step was to construct a δ homolog. The “dT2A” homolog [20] was chosen for this analysis as it has been successfully used to replicate biomarker findings across these cohorts. Regardless, dT2A’s target IADL indicator varies across the cohorts. ADNI uses the FAQ to assess IADL [35], TARCC uses the Lawton Brody IADL measure [34].

Next, we constructed the latent INFLAMMATION construct. INFLAMMATION was previously described as a reflective factor [21]. However, for this application a “formative” approach was used. The formative approach captures the variance shared by the panel of blood-based biomarkers with no residual and facilitates later steps in the algorithm. Formative and latent factors are mathematically interchangeable but might be interpreted differently [39].

Next, we regressed INFLAMMATION onto dT2A. This produces a cognitive residual, “CR”, representing **all** the variance in δ (i.e., in dementia severity) not attributable to INFLAMMATION. Any and all competing influences on dT2A are captured in that residual and so there is no need to adjust INFLAMMATIONS’s effect on dT2A for any particular dementia-related covariate.

Next, we correlated dT2A with CR. A LOI is drawn through their scatterplot rather than a regression. If INFLAMMATION had no effect on dT2A then, CR would be identical to dT2A and the entire sample would be plotted along the LOI. Visual inspection of the scatterplot can test that null hypothesis. Cases presenting off the LOI have demonstrably altered dementia severity after INFLAMMATION’s unique effect is factored out. If CR improves relative to dT2A then INFLAMMATION’s effect was adverse and the case can be said to be “Afflicted” by INFLAMMATION. Otherwise, they are “Resilient”. Because both dT2A and CR are empirically estimable as the sum of weighted dT2A indicators, affliction class can be assigned to an individual solely on the basis of observed psychometric measures and without knowledge of the biomarker of interest. An unknown individual is simply being mapped into the reference cohort’s distribution.

The accuracy of the classification could be impacted by the generalizability of the reference cohort to the population being assessed. Ideally, the reference cohort used to construct the classifier would be similar to the population in which the classifier is to be deployed. ADNI and TARCC are both convenience samples with similar fractions of well-characterized cases of AD, MCI and NC. However, ADNI is limited to NHW, but TARCC is an ethnically diverse cohort that includes a sizable fraction of MA. We and others have previously found significant differences in the serum biomarker profiles of MA and NHW [14,26,40]. To address this, we stratified INFLAMMATION by ethnicity in TARCC and tested for factor equivalence by comparing the CHISQ fit of constrained vs. unconstrained models. Serum protein indicators impacted by ethnicity were dropped, resulting in a factor equivalent version of INFLAMMATION constructed from a subset of the original’s indicators. This ethnicity equivalent construct was revalidated by its association with dT2A. CR did not need adjustment for ethnicity as it represents the influence of all influences on dT2A that are residual to the factor equivalent INFLAMMATION construct, including any residual ethnicity-related influences.

In both cohorts, we validated the affliction class by testing its effect on time to AD conversion from a non-demented baseline diagnosis (i.e., NC or MCI). Time to initial AD conversion was calculated, and the effect tested by survival analysis (i.e., Kaplan-Meier and Cox proportional hazards models) [41]. In TARCC, this analysis was also repeated separately in MA and NHW to confirm affliction class validity in MA who may be less vulnerable to serum INFLAMMATION.

#### SEM

These analyses were conducted in ADNI’s combined sample (N = 1737) and TARCC’s most recent dataset (N = 3497). The analysis was performed using Analysis of Moment Structures (AMOS) software [42]. The maximum likelihood estimator was chosen for these models. Observed indicators were not adjusted for covariates. Co-variances between the residuals were allowed to be estimated if they were significant and improved fit.

#### Missing data

AMOS uses Full information Maximum Likelihood (FIML) methods to address missing data in SEM. It yields unbiased parameter estimates, preserves the overall power of the analysis, and is superior to alternative methods [43]. All other analyses, including correlations, ANOIVA, multiple regression and survival curves were performed in complete cases.

#### Line of identity algorithm

The LOI described above was used to divide the sample into affliction classes. After confirming that dT2A was positively scaled relative to CDR-SB, participants presenting below the LOI were labeled as Afflicted” by INFLAMMATION. Participants presenting at or above the LOI were labeled as “Resilient”. To validate those classifications, both groups were contrasted on their CDR-SB scores, INFLAMMATION scores, and observed indicator biomarker levels [by one way Analysis of Variance (ANOVA)]. We also tested the effect of LOI affliction class on time to AD conversion from non-demented baseline diagnoses (NC + MCI).

We then replicated the entire analysis in TARCC /serum. TARCC is an ethnically diverse subset, and so we used the ethnicity equivalent INFLAMMATION construct from a subset of the original’s indicators. The effect of LOI affliction class on time to AD conversion was tested separately in NHW and MA.

#### Fit indices

One advantage of our SEM approach is that the validity of structural models can be assessed by certain statistical tests. A non-significant chi-square signifies that the data are consistent with the model [44]. However, the ratio of the chi-square to the degrees of freedom in the model is also of interest. A CMIN/DF ratio < 5.0 suggests an adequate fit to the data [45]. The comparative fit index (CFI), with values ranging from between 0 and 1, compares the specified model with a model of no change [46]. CFI values below 0.95 suggest model misspecification. Values of 0.95 or greater indicate adequate to excellent fit. A root mean square error of approximation (RMSEA) of 0.05 or less indicates a close fit to the data, with models below 0.05 considered “good” fit, and up to 0.08 as “acceptable” [47]. All three should be simultaneously considered to assess the model’s fit to the data.

## Results

Descriptive statistics are presented in Tables 1 and 2. Adversely impacted cases were significantly more likely to be ε4 carriers, more impaired on multiple cognitive measures and exhibited higher CDR-SB scores in both cohorts. Adversely impacted cases were significantly less well educated in both cohorts. They had higher levels of informant-reported disability and were more likely to be depressed, or female in TARCC, but not in ADNI.

**Table 1:** Descriptive statistics in ADNI.

Variable	All Cases N = 1737 Mean (SD)	“Resilient” Above the LOI n = 1091 Mean (SD)	“Afflicted” Below the LOI n = 646 Mean (SD)	t (1735)	*p* **
**Age** (years)	73.77 (7.20)	72.77 (7.33)	75.46 (6.93)	7.67	<0.001
**Animals**	17.15 (5.93)	17.96 (5.62)	16.60 (6.69)	1.92	0.055
**APOE e4 allele** (%)	56.8	48.58	70.78	6.78	<0.001
**BNT**_**60**_	25.97 (4.51)	27.23 (3.24)	23.84 (5.45)	-16.23	<0.001
**EDUC (years)**	15.91(2.86)	16.31 (2.66)	15.22 (3.05)	-7.79	<0.001
**FAQ**	4.26 (6.26)	1.86 (3.71)	8.32 (7.47)	24.00	<0.001
**GDS**_**30**_	1.42 (1.40)	1.29 (1.36)	1.65 (1.43)	5.20	<0.001
**Gender** (%♂)	55.09	53.80	57.28	1.41	0.16
**MMSE**	27.17 (2.67)	28.41 (1.78)	25.09 (2.62)	-31.43	<0.001
**Trails B (sec)**	122.23 (75.78)	99.68 (59.15)	160.33 (85.03)	17.48	<0.001
**WMS LMI** *	9.28 (4.83)	11.25 (4.27)	5.96 (3.79)	-25.97	<0.001
**WMS LM II** *	7.07 (5.33)	3.28 (4.92)	9.32 (3.54)	-27.27	<0.001

*Scaled scores.

** by t test across LOI class.

Animals = Animal Naming, BNT = Boston Naming Test; CDR = Clinical Dementia Rating scale; EDUC = education; FAQ = Functional Abilities Questionnaire; GDS = Geriatric Depression Scale; MMSE = Mini-mental State Exam; SD = standard deviation; Sec = seconds, WMS LM I = Weschler Memory Scale: Immediate Logical Memory; WMS LM II = Weschler Memory Scale: Delayed Logical Memory.

**Table 2:** Descriptive statistics in TARCC.

Variable	All Cases N = 3497 Mean (SD)	“Resilient” Above the LOI n = 1937 Mean (SD)	“Afflicted” Below the LOI n = 1560 Mean (SD)	t (3495)	*p* **
**Age** (years)	70.80 (9.56)	68.08 (9.12)	74.11 (9.02)	19.41	<0.001
**Animals**	14.48 (5.57)	16.98 (4.64)	11.37 (5.04)	-34.20	<0.001
**APOE e4 allele (%)**	45.70	34.35	59.32	11.59	<0.001
**BNT**_**60**_ *	7.90 (4.22)	8.82 (4.31)	6.76 (3.82)	-14.84	<0.001
**EDUC** (years)	13.30 (4.28)	13.46 (4.18)	13.11 (4.39)	-2.41	0.02
**GDS**_**30**_	5.58 (5.23)	5.29 (5.20)	5.97 (5.26)	3.62	<0.001
**Gender** (%♂)	38.41	31.92	41.51	-3.39	<0.001
**Hispanic (%MA)**	35.70	40.86	29.43	-7.01	<0.001
**IADL**	10.64 (4.89)	8.23 (1.90)	13.64 (5.74)	38.93	<0.001
**MMSE**	25.58 (4.70)	28.09 (2.17)	22.46 (5.12)	-43.82	<0.001
**Trails B** *	7.49 (3.90)	8.87 (3.52)	5.73 (3.65)	-25.73	<0.001
**WMS LMI** *	7.92 (4.09)	9.77 (3.48)	5.62 (3.58)	-32.63	<0.001
**WMS LM II** *	8.23 (4.39)	10.43 (3.42)	5.50 (3.90)	-39.80	<0.001

*Scaled scores.

** by t test across LOI class.

Animals = Animal Naming, BNT = Boston Naming Test; CDR = Clinical Dementia Rating scale; EDUC = education; FAQ = Functional Abilities Questionnaire; GDS = Geriatric Depression Scale; IADL = Instrumental Activities of Daily Living; MA = Mexican American; MMSE = Mini-mental State Exam; SD = standard deviation; Sec = seconds, WMS LM I = Weschler Memory Scale: Immediate Logical Memory; WMS LM II = Weschler Memory Scale: Delayed Logical Memory.

### ADNI results

In ADNI, dT2A was significantly inversely related to its cognitive indicators and positively with FAQ and CDR-SB. In ADNI /plasma, the INFLAMMATION factor had excellent fit (CHISQ = 8.12, df = 7; CFI = 0.977, RMSEA = 0.01). However, not all of INFLAMMATION’s reflective indicators loaded significantly on the formative construct. A2M, IL -13, PPP, and SAP had significant loadings ranging from r = 0.62 (A2M) to r = 0.31 (SAP), all p <0.04. TNFa’s moderately strong loading exhibited a trend (r = 0.27, p = 0.057). The others, IL-3, IL-10, PRL, THPO, and vWF, did not load significantly independently in the ADNI model and were removed. It should not be surprising that a significant indictor of a reflective factor loses significance in a formative model. That suggests interactions among the indicators such that one’s effect is fully attenuated (in the formative model) by one or more of the other indicators.

Plasma INFLAMMATION correlated moderately (in complete cases) with dT2A (r = 0.45, p <0.001), and with CDR-SB (r = 0.37, p <0.001). These positive associations suggest that higher INFLAMMATION scores are adverse.

**Fig 1:**
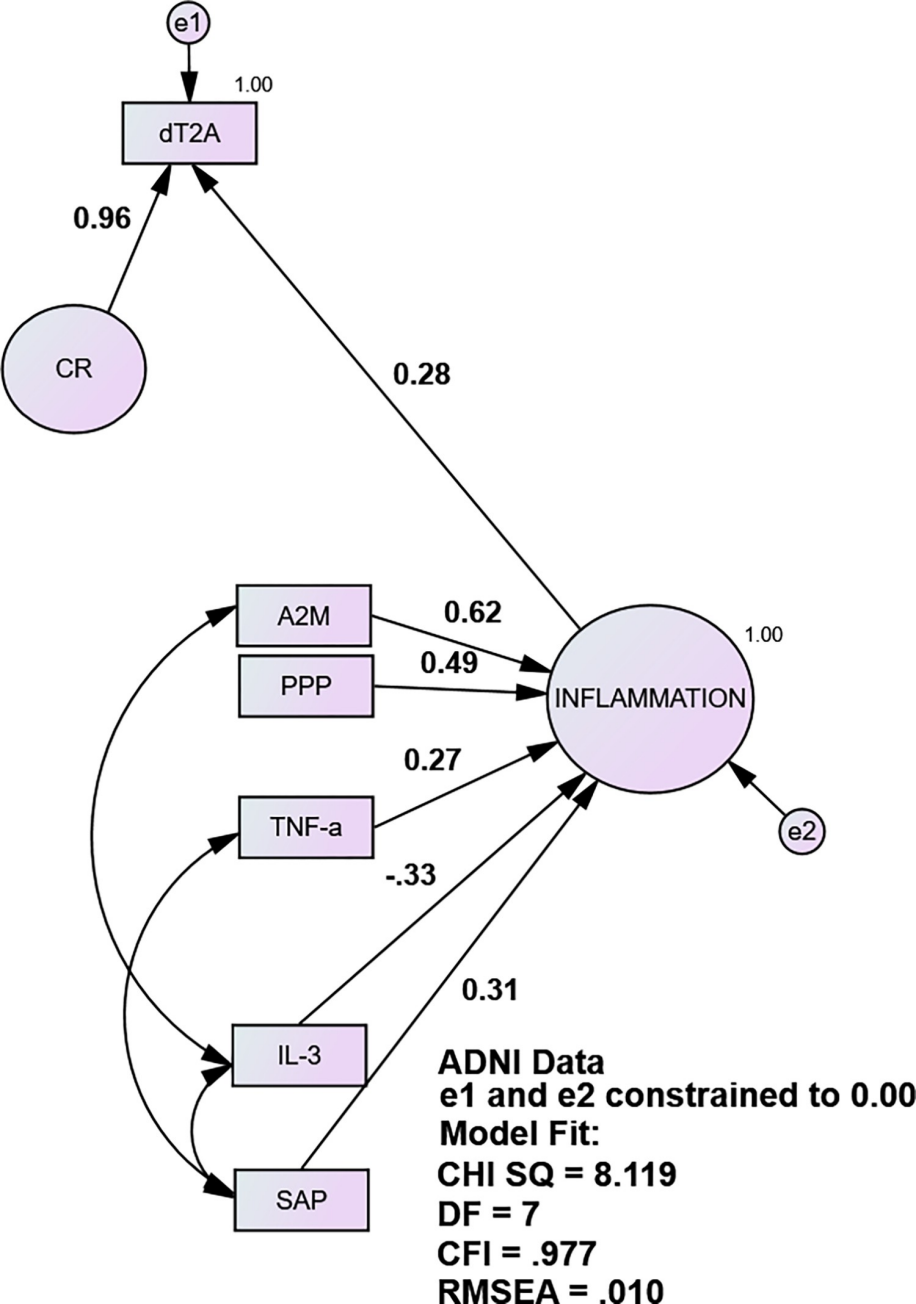
Cognitive Residual factor (CR) (ADNI).

Fig 1 demonstrates the derivation of the CR factor via regression in SEM. The residuals e1 and e2 are constrained to zero variance. e2 is constrained to define the formative INFLAMMATION factor. e1 is constrained to force dT2A’s residual variance into CR, i.e., the variance residual to INFLAMMATION’s effect.

CR sequesters the variance in observed dementia severity (i.e., dT2A) due to any and all influences, excepting INFLAMMATION. Table 3 validates CR. Together, it and INFLAMMATION explain 100% of the variance in clinician-rated CDR-SB.

**Table 3:** CR’s validation in plasma /ADNI.

N = 3497	Dependent Variable: dT2A: R = 1.00 R^2^ = 1.00 Adjusted R^2^ = 1.00; F (2, 1734) = 651E15, p<0.001					
	b*	SE of b*	b	SE of b	t (1734)	*p*
**Intercept**			-2.625	0.000	-1.911E+08	<0.001
**INFLAMMATION**	0.175	0.000	0.227	0.000	1.915E+08	<0.001
**CR**	0.935	0.000	0.960	0.000	1.020E+09	<0.001

CR = Cognitive Residual; SE = standard error.

**Fig 2:**
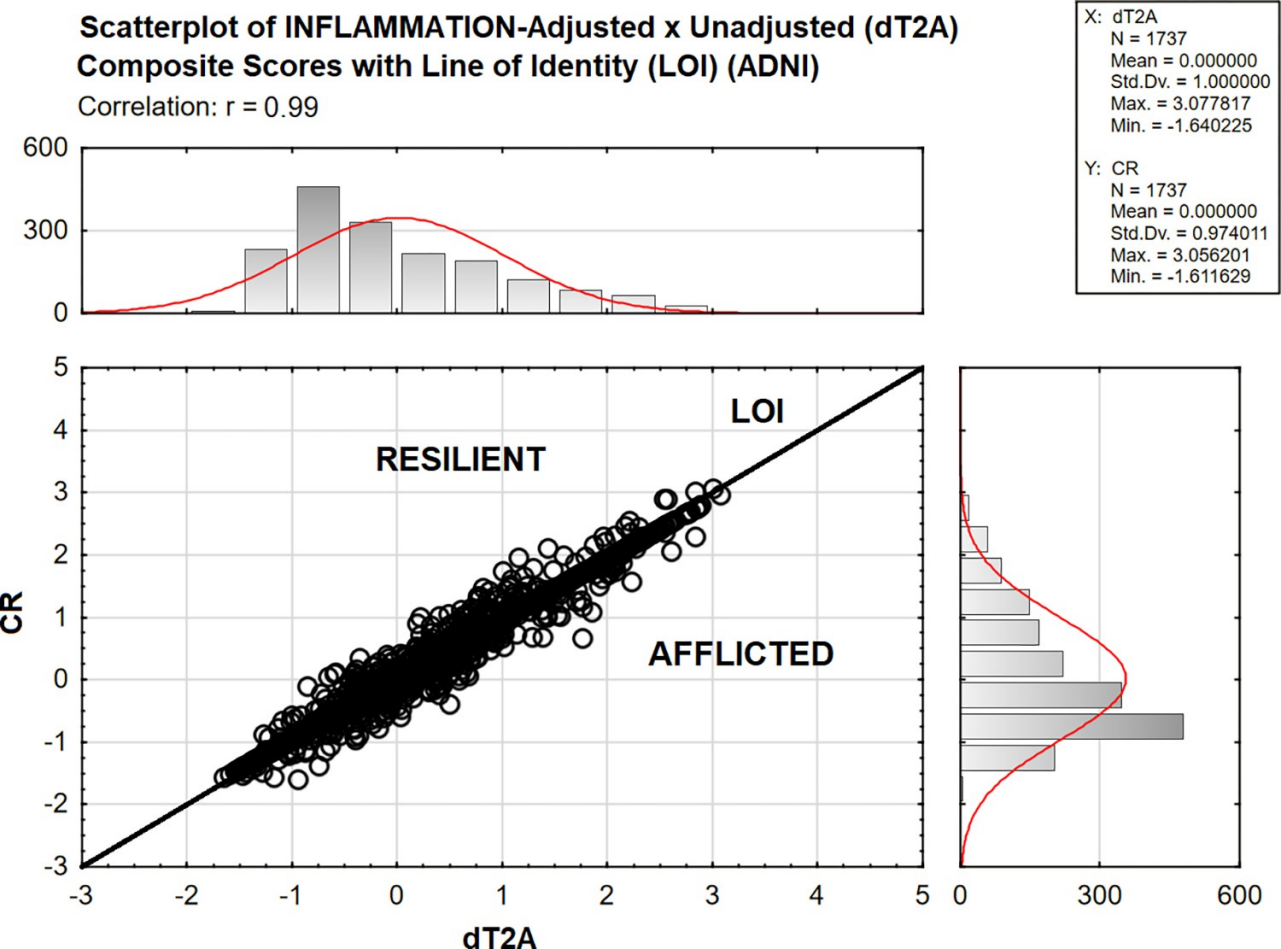
ADNI LOI analysis.

Fig 2 presents the ADNI LOI analysis. The adjusted (i.e., CR) and unadjusted (i.e., dT2A) composites were strongly correlated (i.e., r = 0.99, p <0.001). Because dT2A is positively associated with CDR-SB, higher scores are more adverse. Cases with CR scores below the LOI have relatively improved dementia severity after INFLAMMATION’s unique effect is accounted for. They are “Afflicted”. The remaining cases are “Resilient” to the effects of inflammation as measured by the INFLAMMATION construct in plasma.

N = 1560 /3497 (44.61%) of ADNI’s subjects were adversely impacted by INFLAMMATION. Afflicted cases presented across dT2A’s entire range, but especially at lower dT2A scores, i.e., at less demented stages of dementia’s evolution.

**Fig 3:**
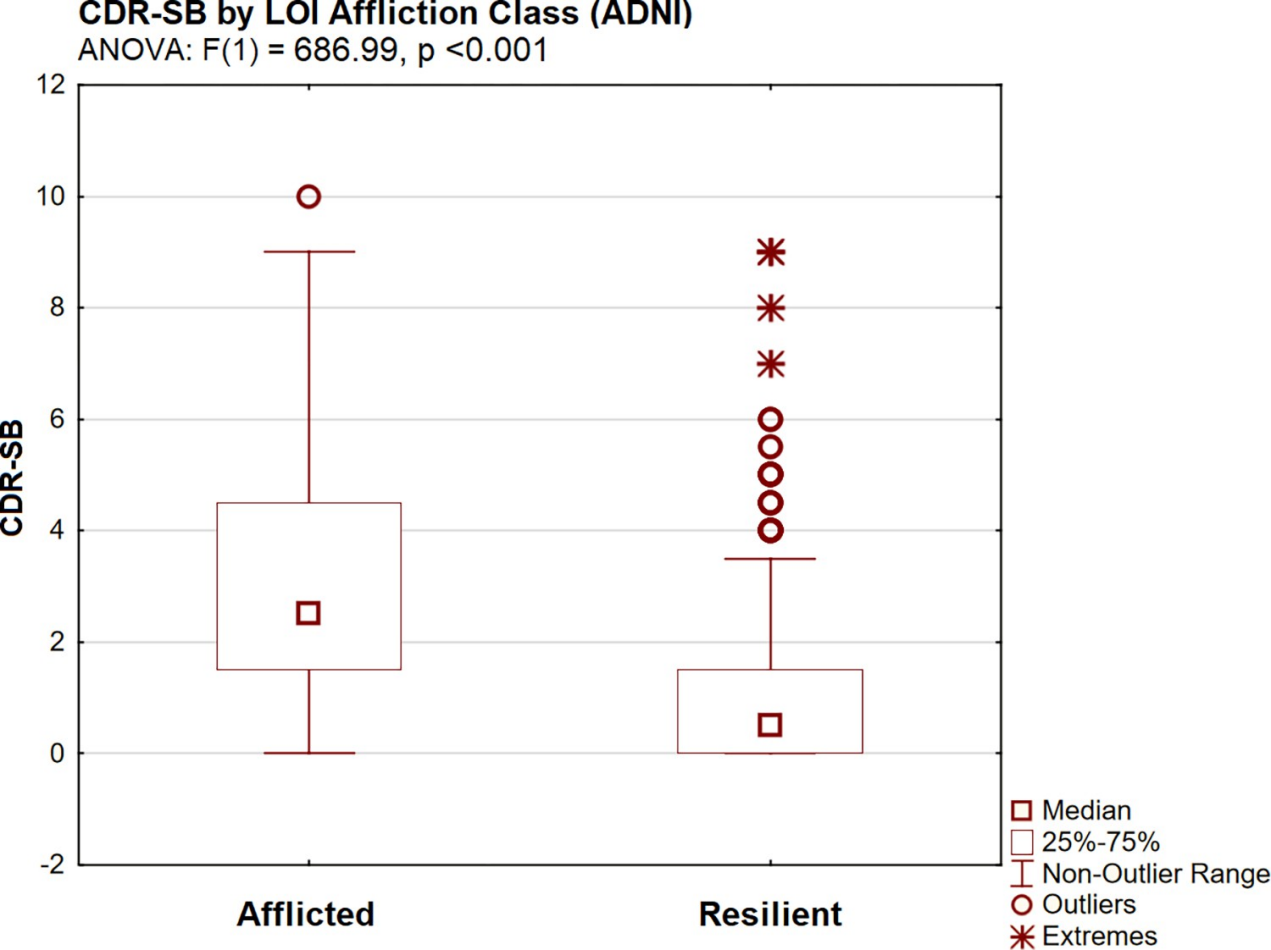
CDR-SB by LOI group (ADNI).

**Fig 4:**
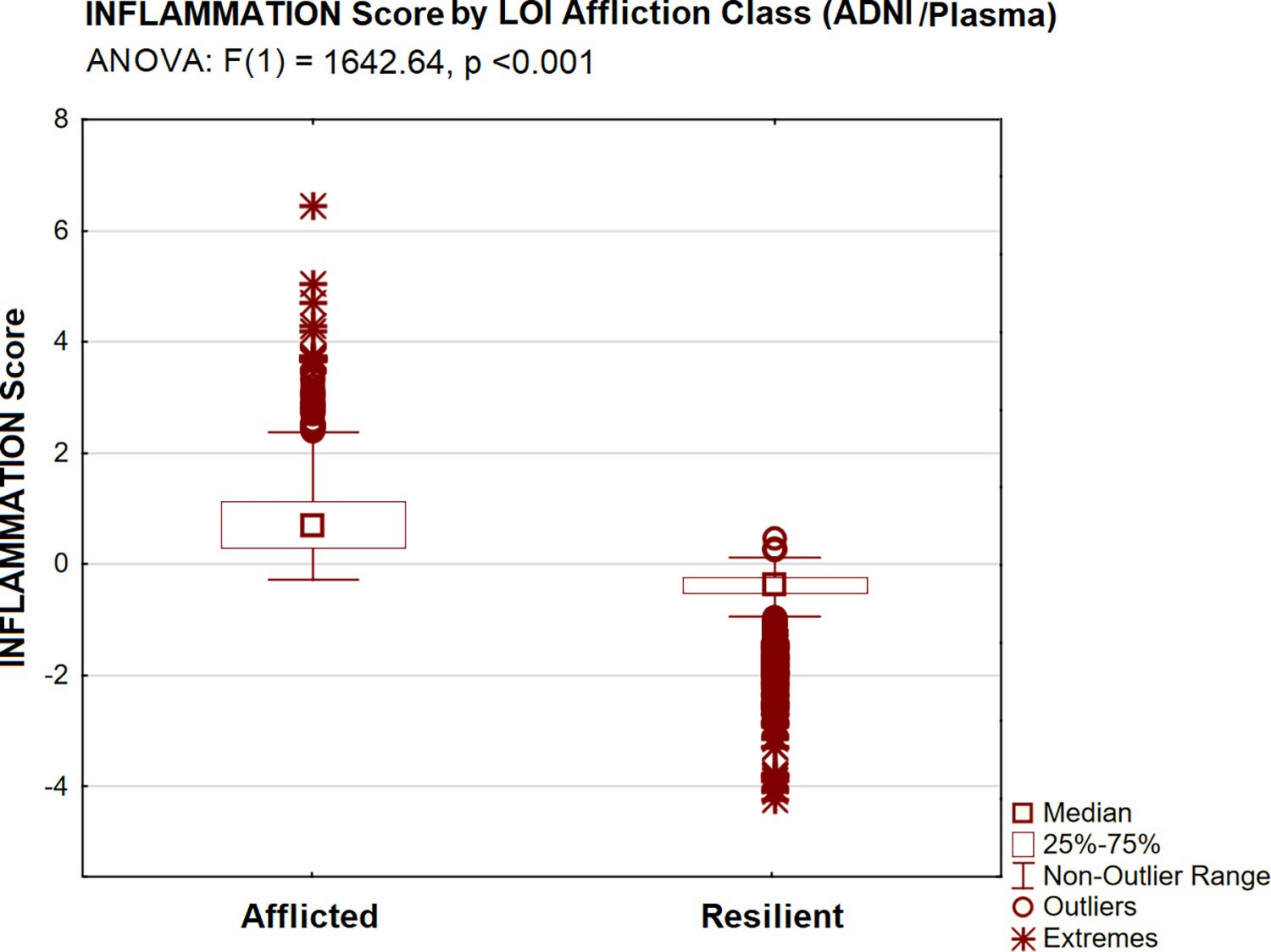
INFLAMMATION by LOI group (ADNI /plasma).

CDR-SB scores were significantly higher in the afflicted LOI class [by ANOVA: F(1) = 686.99, p <0.001] (Fig 3). INFLAMMATION scores (in plasma) were significantly higher (adverse) in afflicted subjects [by ANOVA: F(1) 1642.64, p <0.001] (Fig 4), as were observed levels of each of INFLAMMATION’s serum protein indicators (i.e., by Tukey’s HSD, all p <0.001).

**Fig 5:**
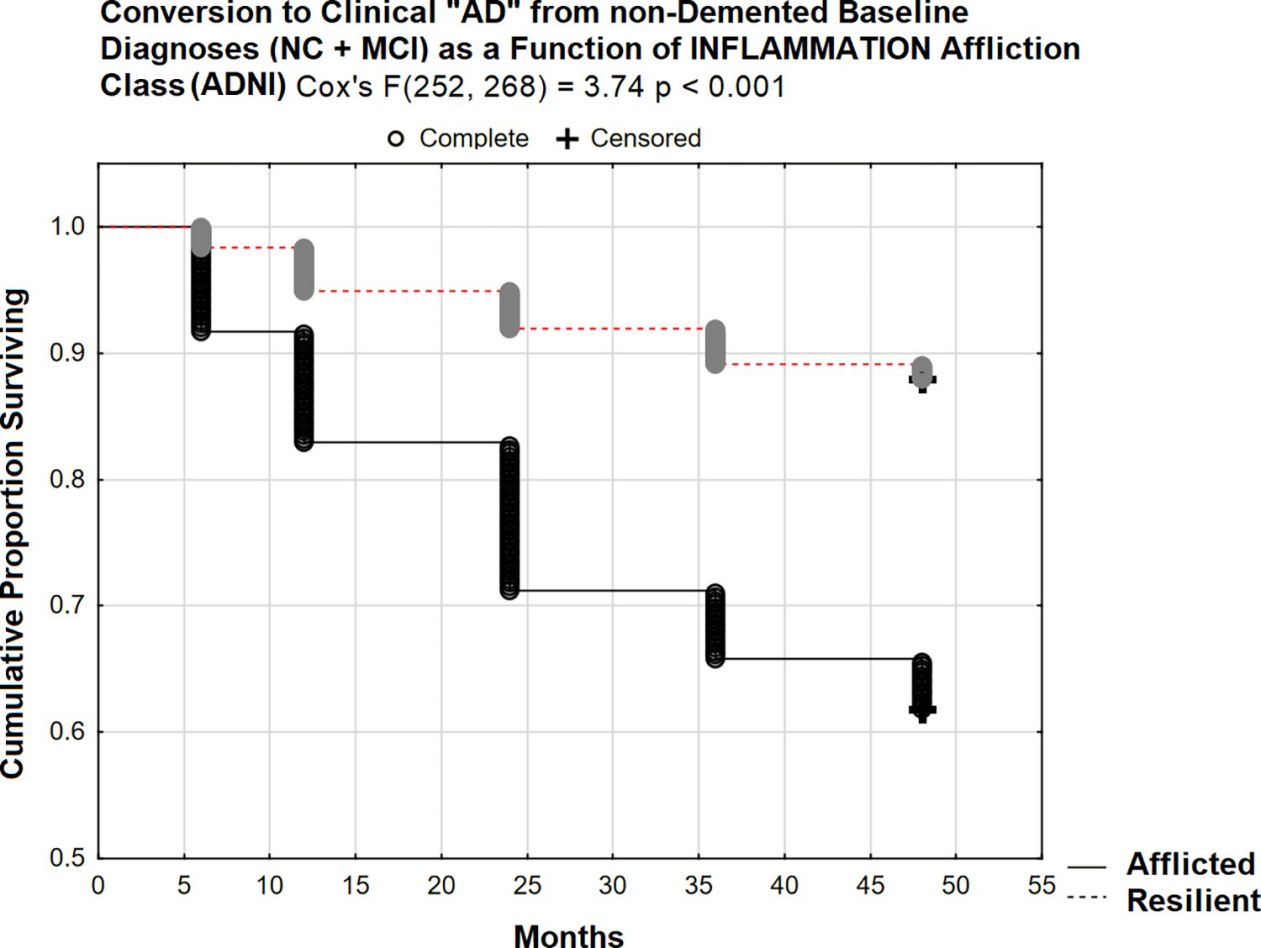
48 month prospective conversion to clinical “AD” from non-demented states (ADNI).

Affliction class had a significant effect on four year (48 month) prospective conversion to clinical “AD” from non-demented states (Cox’s F(252, 268) = 3.74 p < 0.001; Fig 5).

### TARCC results

As previously reported for TARCC NHW [19], dT2A in the entire sample had excellent fit [i.e., CHI SQ = 52.52 (7), p < 0.001; CFI = 0.996; RMSEA = 0.043] and correlated strongly with the CDR-SB (r = 0.87, p <0.001). dT2A was significantly inversely related to all of its cognitive indicators and positively with IADL and CDR-SB.

The formative serum INFLAMMATION model was significantly indicated by all eight serum proteins (all p <0.02). However, only four exhibited factor equivalence across ethnicity (i.e., A2M, PPP, TNFa and vWF). Table 4 presents the change in CHISQ fit (ΔCHISQ) in constrained v. progressively unconstrained models. The constrained ethnicity-equivalent INFLAMMATION factor was used in subsequent analyses.

**Table 4:** Change in CHISQ (ΔCHISQ) in INFLAMMATION models stratified on ethnicity.

Model	CHISQ (df)	ΔCHISQ (Δdf)	*p*
**Constrained:**	465.80 (77)		
**A2M released**	465.80 (78)	0.00 (1)	p = 0.99
**PPP released**	462.00 (76)	3.80 (1)	*p* >0.05
**TNFa released**	461.91 (75)	0.09 (1)	*p* >0.75
**vWF released**	461.86 (74)	0.05 (1)	*p* >0.75

A2M = Alpha-2 macroglobulin; CHISQ = Chi Square; ΔCHISQ = change in Chi Square; df = degrees of freedom; Δdf = change in degrees of freedom; PPP = pancreatic polypeptide; TNFa = Tumor necrosis factor alpha.

The ethnicity equivalent INFLAMMATION factor had acceptable fit (CHISQ = 8.84, df = 1; CFI = 0.956, RMSEA = 0.047) in TARCC /serum. All indicators had significant loadings ranging from r = 0.59 (vWF) to r = 0.39 (SAP), all p <0.001.

INFLAMMATION correlated moderately with dT2A (r = 0.56, p <0.001), and with CDR-SB (r = 0.48, p <0.001), similarly to the formative plasma INFLAMMATION factor in ADNI subjects (above) and to what has been reported for the reflective serum INFLAMMATION factor in TARCC participants (in NHW) [21]. These positive associations suggest that higher INFLAMMATION scores are adverse.

**Fig 6:**
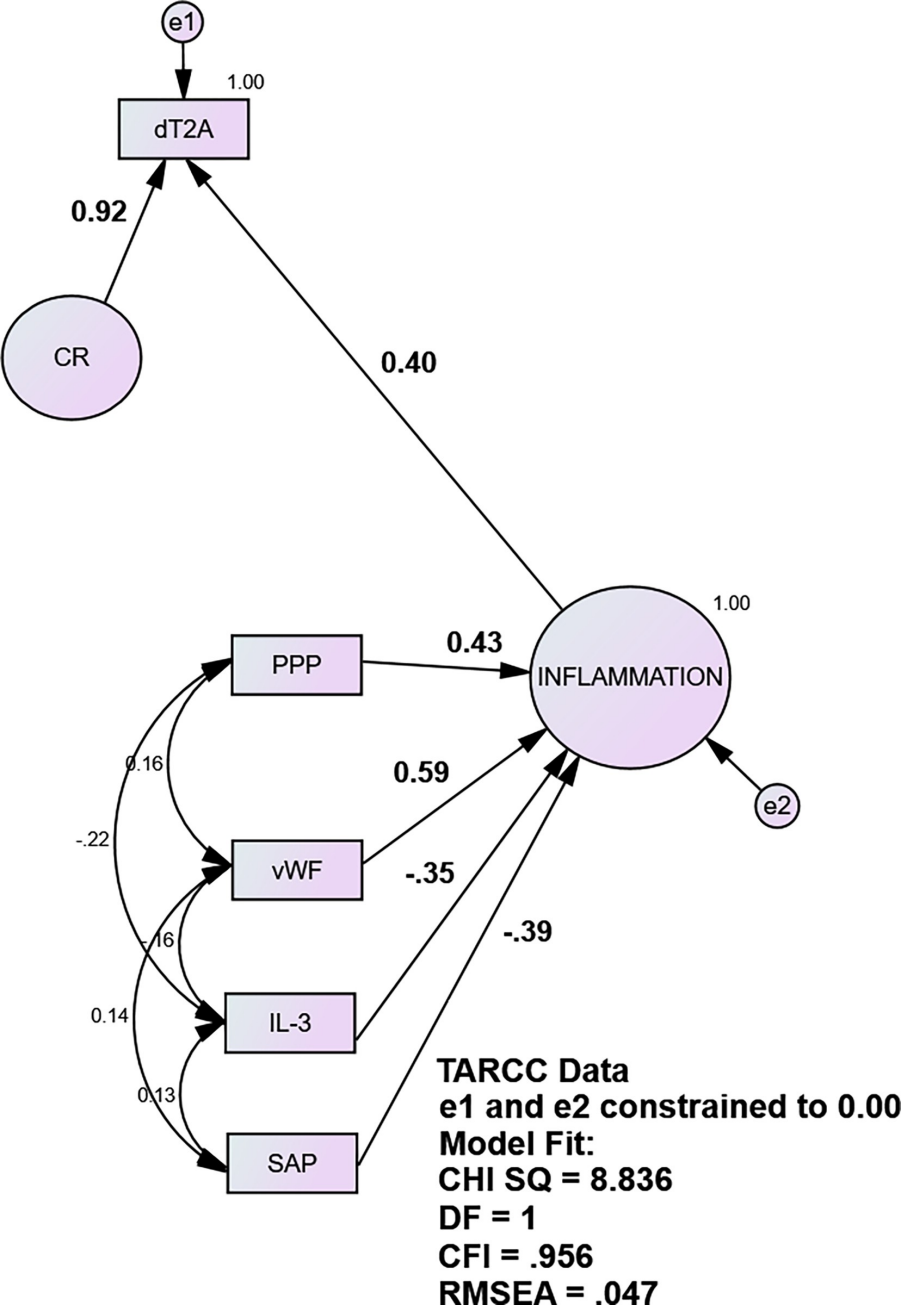
Cognitive Residual factor (CR) (TARCC).

Fig 6 demonstrates the derivation of CR in TARCC. The residuals e1 and e2 are again constrained to zero variance. Table 5 validates CR. Together, it and INFLAMMATION explain 100% of the variance in TARCC clinician-rated CDR-SB.

**Table 5:** CR’s validation in serum /TARCC.

N = 3497	Dependent Variable: dT2A: R = 1.00 R^2^ = 1.00 Adjusted R^2^ = 1.00; F(2,3494) = 131E16, p<0.001					
	b*	SE of b*	b	SE of b	t (3494)	*p*
**Intercept**			-0.047	0.00	-7.346E+07	<0.001
**INFLAMMATION**	0.200	0.00	0.267	0.00	2.961E+08	<0.001
**CR**	0.902	0.00	0.941	0.00	1.334E+09	<0.001

CR = Cognitive Residual; SE = standard error.

**Fig 7:**
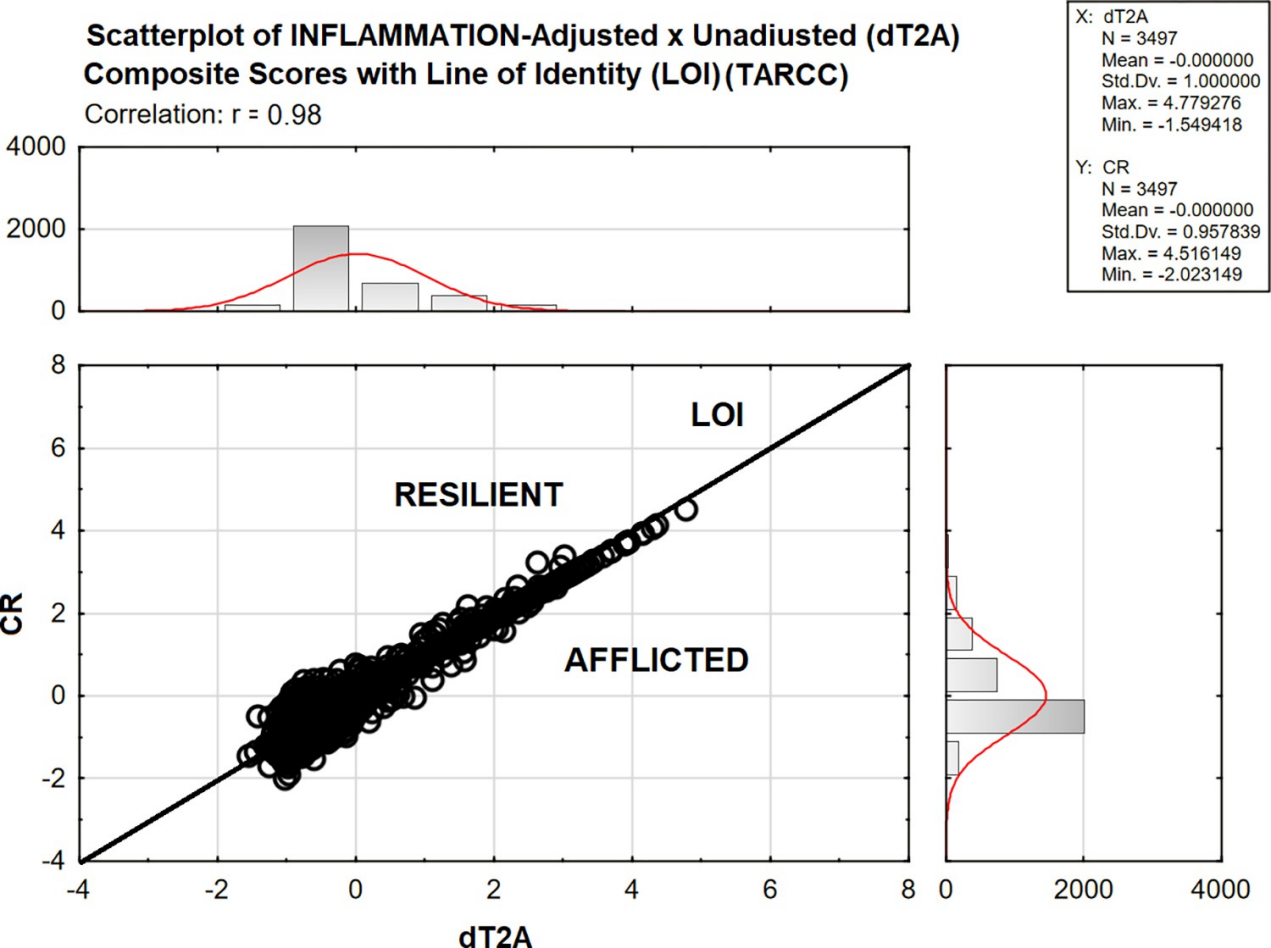
TARCC LOI analysis.

Fig 7 presents the TARCC LOI analysis. The adjusted (i.e., CR) and unadjusted (i.e., dT2A) composites were again strongly correlated (i.e., r = 0.98, p <0.001). Because dT2A is positively associated with CDR-SB, higher scores are more adverse. Cases with CR scores below the LOI have relatively improved dementia severity after INFLAMMATION’s unique effect is accounted for. They are “Afflicted”. The remaining cases are “Resilient” to the effects of INFLAMMATION, as measured by an ethnicity-equivalent construct in serum.

N = 1560 /3497 (44.61%) of TARCC’s subjects were adversely impacted by INFLAMMATION. N = 1096 /2215 (49.48%) of NHW subjects were afflicted. 457 /1230 (37.16%) of MA were afflicted. As in ADNI, afflicted cases presented across dT2A’s entire range, but especially at lower dT2A scores, i.e., at less demented stages of dementia’s evolution.

**Fig 8:**
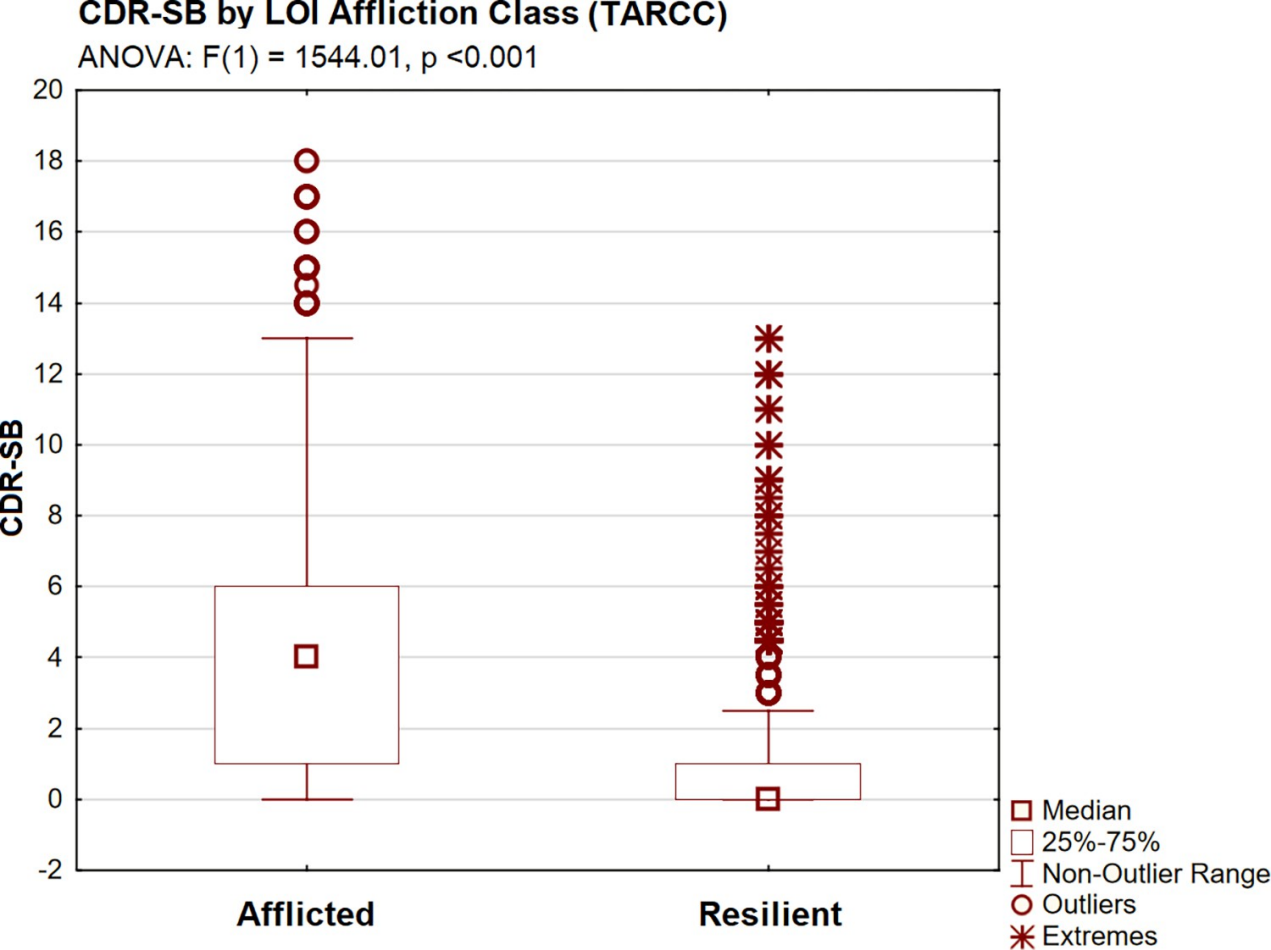
CDR-SB by LOI group (TARCC).

**Fig 9:**
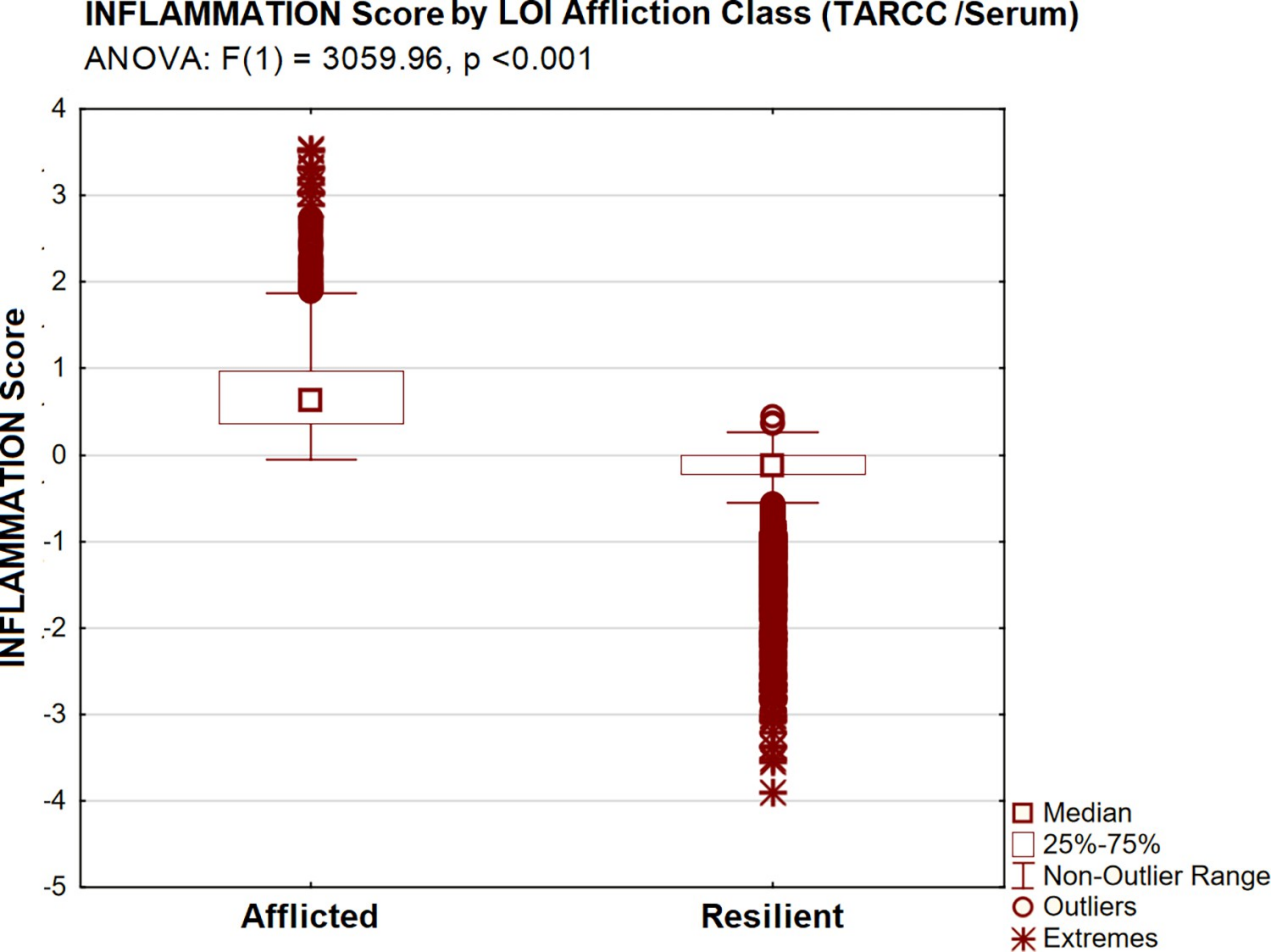
INFLAMMATION by LOI group (TARCC /serum).

CDR-SB scores were significantly higher in the Afflicted LOI class [by ANOVA: F(1) = 1544.01, p <0.001] (Fig 8). Ethnicity-equivalent INFLAMMATION scores (now in serum) were again significantly higher (adverse) in Afflicted TARCC subjects [by ANOVA: F(1) = 3059.96, p <0.001] (Fig 9), as were adverse observed levels of all four of its serum protein indicators (i.e., by Tukey’s HSD, all p <0.001).

**Fig 10:**
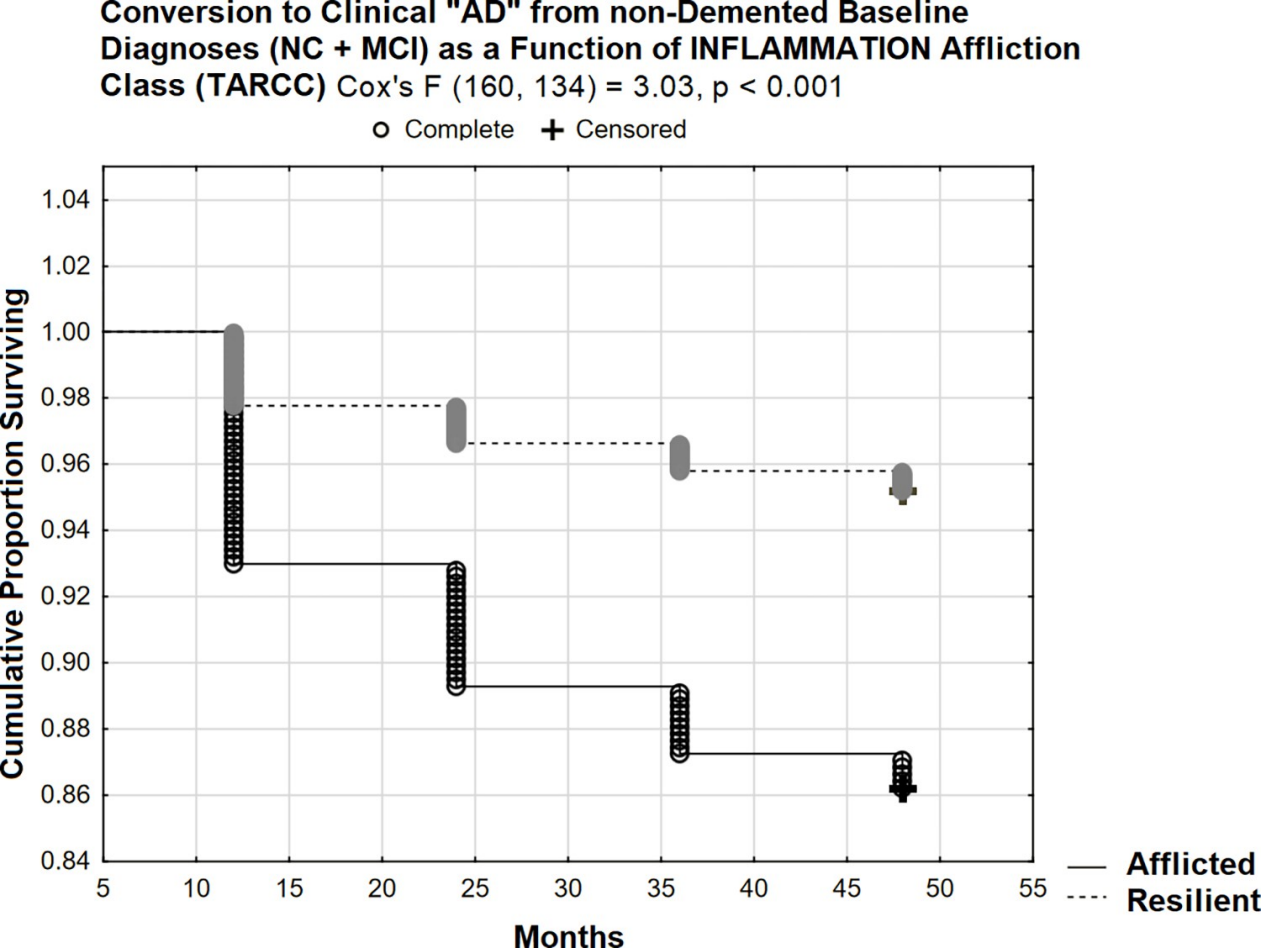
48 month prospective conversion to clinical “AD” from non-demented states (TARCC).

INFLAMMATION was significantly associated with CDR-SB independently of several other dementia risks (Table 6). Affliction class explains additional variance in CDR-SB and selectively attenuates INFLAMMATION’s effect (Table 7). Affliction class also had a significant effect on prospective conversion to clinical “AD” from non-demented states (Cox’s F (160, 134) = 3.03, p < 0.001) in TARCC’s entire sample (Fig 10).

**Table 6:** INFLAMMATION explains variance in CDR-SB (in TARCC’s bi-ethnic sample) independently of other dementia risks.

N = 2942	Dependent Variable: CDR-SB, R = 0.57, R^2^ = 0.33, Adjusted R^2^ = 0.327; F (6,2935) = 239.44, p<0.001					
	b*	SE of b*	b	SE of b	t (2935)	*p*
**Intercept**			-6.374	0.436	-14.625	<0.001
**Age**	0.283	0.016	0.093	0.005	17.950	<0.001
**GENDER**	0.019	0.016	0.119	0.099	1.210	0.227
**EDUCATION**	0.093	0.016	0.066	0.012	5.672	<0.001
**APOE4**	0.190	0.015	0.953	0.077	12.332	<0.001
**GDS**	0.086	0.016	0.051	0.009	5.440	<0.001
**INFLAMMATION**	**0.351**	0.016	1.407	0.064	21.858	<0.001

AD = Alzheimer’s Disease; APOE4 = the number of apolipoprotein E ε4 alleles; CDR-SB = Clinical Dementia Rating scale “Sum of Boxes”; GDS = Geriatric Depression Scale; LOI = Line of Identity; SE = standard error; TARCC = Texas Alzheimer’s Research and Care Consortium.

**Table 7:** LOI class adds variance to CDR-SB (in TARCC’s bi-ethnic sample) and attenuates INFLAMMATION’s unique effect.

N = 2942	Dependent Variable: CDR-SB, R = 0.63, R^2^ = 0.39, Adjusted R^2^ = 0.390; F (7,2934) = 269.57 p < 0.001					
	b*	SE of b*	b	SE of b	t (2934)	*p*
**Intercept**			-6.009	0.416	-14.462	<0.001
**Age**	0.236	0.015	0.077	0.005	15.306	<0.001
**GENDER**	0.024	0.015	0.154	0.094	1.645	0.100
**EDUCATION**	0.092	0.016	0.065	0.011	5.88	<0.001
**APOE4**	0.150	0.015	0.753	0.075	10.112	<0.001
**GDS**	0.080	0.015	0.047	0.009	5.332	<0.001
**INFLAMMATION**	0.130	0.020	0.523	0.080	6.572	<0.001
**INFLOI**	0.353	0.020	2.196	0.126	17.398	<0.001

**Nb R**^**2**^ **increase relative to Table 6 and INFLAMMATION’s attenuation.**

AD = Alzheimer’s Disease; APOE4 = the number of apolipoprotein E ε4 alleles; CDR-SB = Clinical Dementia Rating scale “Sum of Boxes”; GDS = Geriatric Depression Scale; LOI = Line of Identity; SE = standard error; TARCC = Texas Alzheimer’s Research and Care Consortium.

**Fig 11:**
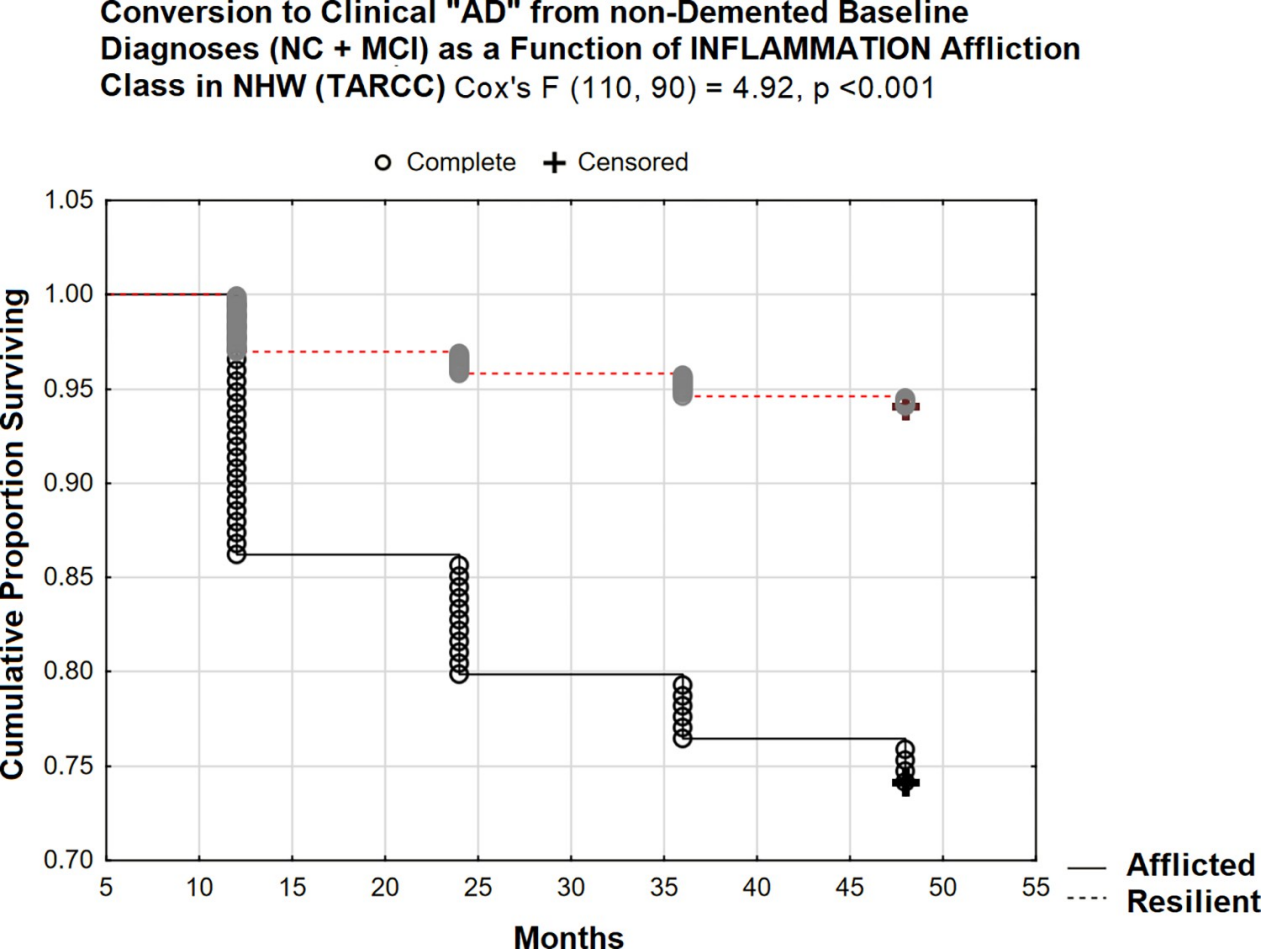
48 month prospective conversion to clinical “AD” from non-demented states in NHW (TARCC).

Regardless, 14% conversion at 48 months in the afflicted class appears less aggressive than in ADNI /plasma. However, in ADNI, INFLAMMATION’s effect is being tested in NHW only (and in plasma). In TARCC NHW (serum), similar results are obtained (Cox’s F (110, 90) = 4.92, p <0.001; Fig 11). 26% of initially non-demented NHW in the afflicted class had converted by 48 months.

**Fig 12:**
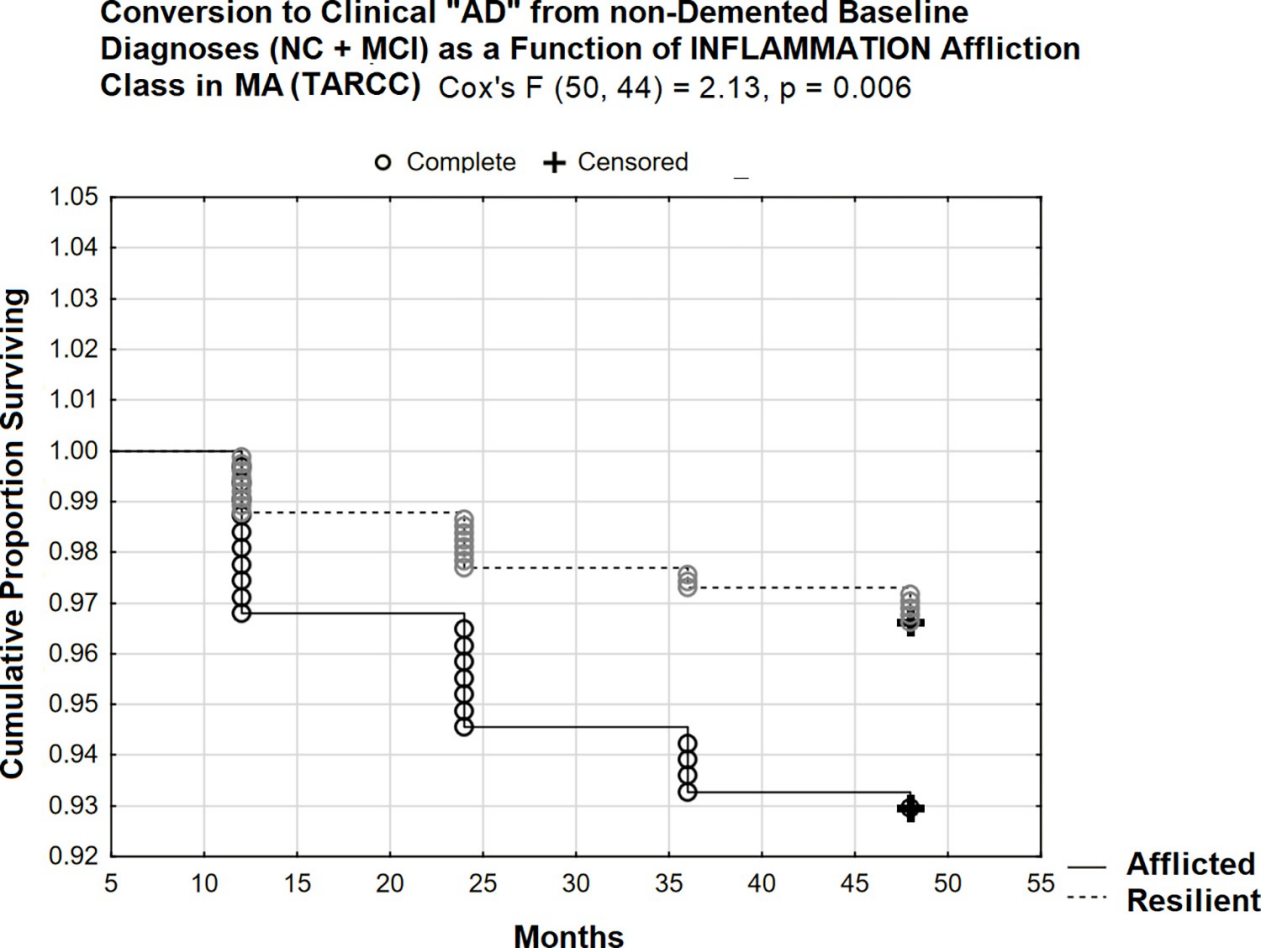
48 month prospective conversion to clinical “AD” from non-demented states in MA (TARCC).

In MA, the effect of affliction by INFLAMMATION is less impactful, yet still significant (Cox’s F (50, 44) = 2.13, p = 0.006; Fig 12). Only 7% in the afflicted class had converted by 48 months. This ethnicity-specific effect cannot be easily attributed to either the biofluid involved, or to the restricted set of ethnicity-equivalent INFLAMMATION indicators given the replication of INFLAMMATION effects in NHW across both cohorts /biofluids.

Finally, LOI adds variance to CDR-SB independently of INFLAMMATION and competing dementia risks. Nb, the incremental R^2^ increase with the addition of affliction class to the model and that only INFLAMMATION’s effect is attenuated. This demonstrates our algorithm’s ability to isolate individual dementing influences from competing risks and the modulation of a biomarker’s effect as a function of affliction class.

## Discussion

These findings recapitulate earlier findings from TARCC and ADNI [20]. INFLAMMATION is related to dT2A across cohorts and biofluids. We also demonstrate by a novel method that its effects span the entire range of dementia severity, i.e., dT2A’s entire distribution, and that its influence is less important in dementia’s later stages. Both findings replicate across cohorts and biofluids.

Some readers might be concerned for possible circularity in δ’s construction being a latent construct derived from observed test scores. However, the TARCC /ADNI raters making their diagnoses were utterly unaware of the subjects’ d-scores, as this is a post-hoc secondary analysis. Moreover, the LOI’s validation is the subject of this analysis, not dT2A’s which has been previously described [20]. The LOI is validated by biomarker effects and prospective conversions to dementia, not diagnostic classifications. It is worth noting that our algorithm might equally have been applied to observed CDR-SB scores, or a latent variable constructed from CDR items. Knowledge of those observed scores would not have compromised the analysis yet dT2A would not have been used at all. Regardless, our use of a δ homolog has three key advantages over the CDR 1) d-scores can be estimated without input from “expert” clinicians with varying skills and experience, 2) δ can be estimated from any reasonably comprehensive cognitive battery and so can be engineered to be assessed remotely, by telephone etc. (i.e., “dTEL” [48]) and 3) d-scores are continuously distributed, even among normal controls and so allow for biomarker associations at any point in dementia’s evolution. The CDR has little or no variance in non-demented persons and is thereby limited in its utility in very early stages of an illness. This last point also speaks to how a rater with knowledge of the CDR score would be unable to estimate δ.

It cannot be determined from this cross-sectional analysis whether the effects of INFLAMMATION wain as dementia evolves or whether the strong selection bias against non-AD dementias in these two convenience samples selects against INFLAMMATION-associated cognitive impairment. This analysis does not address whether INFLAMMATION’s effects on δ might be mediated by AD-specific pathological changes [e.g., central nervous system (CNS) amyloidopathy or tauopathy] or whether it acts independently of them. If independent, then CNS amyloidopathy (e.g., as measured by PET) would be expected to attenuate CR’s association with dT2A and not INFLAMMATION’s.

We have further shown that a psychometric aproach can be used to identify individuals with pre-specified biomarker profiles *without reference to the biomarker(s) in question*. Neither CR nor dT2A reference observed levels of blood-based biomarkers. Thus, they might be estimated in any individual with dT2A’s psychometric indicators and in the absence of biomarker assessment.

Since our approach might be applied to any δ-related biomarker, our algorithm might facilitate the assessment of affliction by imaging and /or CSF biomarkers, but without the need for their expensive, potentially hazardous, difficult to acquire, and /or difficult to perform assessment. Moreover, because δ homologs are largely indifferent to their indicators, LOI classification should be feasible remotely, by telephone, internet, etc. One significant caveat is that since both ADNI and TARCC are convenience samples, it remains to be seen if parameter weights derived from them can be generalized to newly assessed cases.

Our algorithm also identifies individuals who are “resilient” against INFLAMMATION. This should not be surprising. It has long been appreciated that similarly demented cases vary widely in their pathological severity. Many theories have been advanced to explain this phenomenon. However, the field has been hampered by difficulties in assessing “pathological severity” *in vivo* and in accounting for the overdetermined nature of observed dementia severity. Our approach can isolate the effect of a single biomarker of interest and identify individuals at any stage of dementia severity who are resilient to its effect. Future studies could now address the mechanisms of resilience by contrasting affliction classes while adjusting for dementia severity via δ and competing influences through CR.

We must also distinguish our INFLAMMATION construct from the so-called “systemic inflammation” attributed to *individual* blood-based proteins in recent analyses of individual biomarker effects. The INFLAMMATION construct is indicated by both pro- and anti-inflammatory proteins. However, it explains only a fraction of their observed variance. The residual variance in any single biomarker or subset of INFLAMMATION’s indicators is orthogonal to INFLAMMATION and may lead to independent effects (e.g., in other organs). The latent INFLAMMATION construct integrates the shared effect of all of its indicators and is best thought of as a systems level biomarker. AD has recently been proposed to be a systems network disorder involving innate immunity [49], although INFLAMMATION has not been shown to effect changes in dementia severity via AD-specific pathological changes. Inflammation as measured by INFLAMMATION may be an AD-independent determinant of dementia severity in AD and other dementing illnesses.

Replications of biomarker findings across cohorts and biofluids have been notoriously difficult to achieve. We believe the success of our approach derives from a latent variable’s inherent resistance to its indicators’-specific assessment biases, and to the increased biological validity of integrated biomarker effects (i.e., system effects) over the effects of individual proteins. Also, the dT2A construct itself was engineered specifically to facilitate replications between TARCC and ADNI [20] and we have achieved similar replications for both INFLAMMATION itself, and the blood-based protein mediators of the Age-specific effect on δ [21,22].

However, those replications were achieved by restricting the analyses to NHW. Our past experience suggests there may be significant cross-ethnicity differences between MA and NHW with regard to their blood-based biomarker profiles [14,26,40]. Cross-ethnic biomarker differences have been reported in TARCC for genetic markers [50] including δ’s genetic markers [51]. Here, we have demonstrated cross-ethnic differences in INFLAMMATION’s impact on prospective conversion to clinical dementia from non-demented states. Since the conversion risk in afflicted NHW are comparable across both cohorts, biofluids and INFLAMMATION constructs, it seems most likely that these are truly ethnicity-specific differences in inflammation’s impact. This issue deserves further investigation.

## Conclusions

Biomarker-specific psychometric classifiers can be constructed by an LOI approach applied to reified δ homologs. Our inflammation-specific classifier selects individuals with pre-specified biomarker profiles and predicts conversion to “AD” across cohorts, biofluids, and ethnicities. This algorithm might be applied to any dementia-related biomarker making the psychometric estimation of individual biomarker effects feasible without biomarker assessment. Our approach also distinguishes individuals resilient to individual biomarker effects allowing for more accurate prediction and precision intervention.

## Abbreviations

Abbreviation: Definition
A2M: A2 macroglobulin
AD: Alzheimer’s Disease
ADNI: Alzheimer’s Disease Neuroimaging Initiative
ADRDA: Alzheimer’s Disease and Related Disorders Association
AMOS: Analysis of Moment Structures software
Animals: Animal Naming
ANOVA: Analysis of Variance
APOE: Apolipoprotien
AUC: Area under the receiver operating characteristic curve
BNT: Boston Naming Test
CDR-SB: Clinical Dementia Rating scale “Sum of Boxes”
CERAD: Consortium to Establish a Registry for Alzheimer’s Disease
CFA: Confirmatory factor analysis
CFI: Comparative Fit Index
CHI SQ: Chi Square
ΔCHI SQ: Change in Chi Square
CSF: Cerebrospinal fluid
DF: Degrees of freedom
ΔDF: Change in Degrees of freedom
EDUC: Education (years)
FAQ: Functional Assessment Questionnaire
FIML: Full information Maximum Likelihood
FSCI: Functionally Salient Cognitive Impairment
GDS: 30 item Geriatric Depression Scale
IADLs: Instrumental Activities of Daily Living
IL-3: Interleukin 3
IL-13: Interleukin 13
IRB: Institutional Review Boards
LMI: Wechsler Logical Memory immediate
LMII: Wechsler Logical Memory delayed
LOI: Line of Identity
MA: Mexican-American
MCI: Mild Cognitive Impairment
MMSE: Mini-mental State Exam
NAPA: National Alzheimer’s Program Act
NC: Normal Controls
NHW: Non-Hispanic White
NINCDS: National Institute for Neurological Communicative Disorders and Stroke
OARS: Older Adults Resources Scale
PCR: Polymerase Chain Reaction
PET: Positron emission tomography
PPP: Pancreatic Polypeptide
PRL: Prolactin
RBM: Rules-Based Medicine
RMSEA: Root Mean Square Evaluative Assessment
ROC: Receiver Operating Characteristic
SAP: Serum Amyloid Protein
SD: Standard Deviation
SEM: Structural Equation Model
TARCC: Texas Alzheimer’s Research and Care Consortium
THPO: Thrombopoietin
TNF-α: Tumor Necrosis Factor
Trails B: Trail Making Test Part B
US: United States
vWF: von Willebrand factor
